# Evaluation of Arterial Stiffness and Its Relation to Innovative Anthropometric Indices in Persian Adults

**DOI:** 10.1155/2023/2180923

**Published:** 2023-01-23

**Authors:** Zahra Ataee, Atena Aghaee, Seyyed Reza Sobhani, Elaheh Ebrahimi Miandehi, Parisa Pirzadeh, Maryam Alinezhad-Namaghi, Saeid Eslami, Sahar Sobhani

**Affiliations:** ^1^Department of Internal Medicine, Faculty of Medicine, Mashhad University of Medical Sciences, Mashhad, Iran; ^2^Nuclear Medicine Research Center, Mashhad University of Medical Sciences, Mashhad, Iran; ^3^Department of Nutrition, Faculty of Medicine, Mashhad University of Medical Sciences, Mashhad, Iran; ^4^Persian Cohort Research Center, Mashhad University of Medical Sciences, Mashhad, Iran; ^5^Community Health Nurse, Department of Nursing and Midwifery, Faculty of Nursing, Mashhad University of Medical Sciences, Mashhad, Iran; ^6^International UNESCO Center for Health–Related Basic Sciences and Human Nutrition, Mashhad University of Medical Sciences, Mashhad, Iran; ^7^Department of Medical Informatics, School of Medicine, Mashhad University of Medical Sciences, Mashhad, Iran

## Abstract

**Background:**

BMI has been evaluated as an old criterion to evaluate obesity in individuals, but it does not assess abdominal obesity and lean mass. We aimed to evaluate the possible relationship of new anthropometric indices (namely, a body shape index (ABSI), the body roundness index (BRI), the visceral adiposity index (VAI), the visceral fat area (VFA), and waist-hip ratio (WHR)), with one of the known critical factors of atherosclerosis, arterial stiffness.

**Methods:**

Overall 5921 individuals were enrolled and were divided into four groups according to BMI. Novel anthropometric parameters including, ABSI, BRI, VAI, VFA, and WHR were calculated. The carotid-femoral pulse wave velocity (cf-PWV) was used to evaluate arterial stiffness. Multiple regression analysis was performed to assess the relationship between cf-PWV and innovative Anthropometric indices.

**Results:**

This study population consisted of 3109 women and 2812 males. In men with overweight, cf-PWV was significantly related to BMI, ABSI, BRI, WC, VAI, VFA, and WHR. However, among men with obesity, cf-PWV was associated with BRI, VAI, and VFA. Among women with overweight, cf-PWV was also related to all mentioned indices except ABSI; although, cf-PWV was only associated with VFA and WHR in women with obesity.

**Conclusion:**

Our results showed that VFA in women and VAI in men are strongly related to arterial stiffness and can be used to identify predictors of vascular disease or organic vascular dysfunction.

## 1. Introduction

Obesity is one of the problems of today's world that has affected almost all societies and leads to significant complications such as increased insulin resistance, hyperlipidaemia, and hypertension [1]. Various studies have shown that one of the types of increased body fat and obesity associated with cardiovascular complications is increased abdominal fat and abdominal obesity [2].

BMI has been evaluated as an old criterion to check obesity in people, but it does not evaluate abdominal obesity and lean mass; therefore, in recent years, newer criteria are used in addition to BMI to evaluate obesity [3]. New indices, namely, a body shape index (ABSI), the body roundness index (BRI), the visceral adiposity index (VAI), the visceral fat area (VFA), and waist-hip ratio (WHR), have been recently proposed as anthropometric measures. ABSI is statistically independent of height, BMI, and WC (waist circumference) and can be better regarded as abdominal obesity [4]. The BRI is another composition index that is based on WC and height [5]. VAI is a sex-specific surrogate indicator of visceral adiposity accumulation and dysfunction, and it is calculated with both common anthropometric (body mass index (BMI) and waist circumference (WC)) and lipidemic (triglycerides (TG) and high-density lipoprotein (HDL) cholesterol) measurements and is independently related with cardiometabolic risk. Also, VAI was proposed as a new anthropometric measure in 2010 [6]. VFA measures the fat that is stored around a number of critical internal organs, including liver, intestine, and pancreas [7]. WHR is one of the markers of abdominal obesity and has shown an improved ability to identify cardiovascular disease [3]. One study showed that VAI was the useful tool in the men population for the assessment of cardiometabolic risk associated with visceral obesity [6]; furthermore, VFA can be used to predict atherosclerotic cardiovascular diseases in both genders [8, 9].

Several studies have indicated that VAI is more strongly associated with cardiovascular risk and metabolic risk [10]. Also, VFA correlated significantly with glucose intolerance, hypertriglyceridemia [11], hypertension, and cardiac dysfunction in obese subjects [12]. Very few studies to date have examined the differences between VFA, VAI, WHR, and other anthropometric indices on identifying hypertension and cardiovascular disease (CVD).

CVD is one of the most important causes of death in the world, and about 50% of the causes of death are caused by noncommunicable diseases [12]. Arteriosclerosis is influenced by various factors such as age, increased blood pressure, inflammation, and hyperlipidaemia [12], and it has been shown in various studies that the increase in arteriosclerosis has a direct relationship with the increase in cardiovascular events [13–15].

Therefore, knowing the risk factors and preventing them and using a safe method to determine the presence of arterial stiffness is very essential. Aortic arterial stiffness, which can be indirectly measured by pulse wave velocity (PWV) reflects changes in arteriosclerosis [16]. PWV is one of the safe, robust, reproducible, and noninvasive methods to evaluate arterial stiffness [17].

Since arterial stiffness is related to cardiovascular events, identifying predisposing factors for arterial stiffness and prevention and treatment of these factors are helpful in preventing cardiovascular events. Also, previous studies in different countries and ethnicities claimed that anthropometric measurements have different prognostic values for arterial stiffness, and the predictive power of anthropometric indices should be identified for different ethnicities [18, 19]. Therefore, in this study, we decided to evaluate the relationship between arterial stiffness and new body composition indices in the Persian cohort study.

## 2. Materials and Methods

### 2.1. Subjects

The PERSIAN Organizational Cohort Study in Mashhad (POCM) was launched in 2017 to investigate lifestyle and risk factors for noncommunicable diseases (especially cardiovascular events). Details of the Persian Cohort Study have been reported formerly [20]. Between August 2017 and May 2022, individuals (aged 30–70 years) were selected to enroll in the present study. A total of 6093 participants qualified for the analyses. Among these 6093 individuals, 172 were excluded; 102 were excluded due to incomplete arterial stiffness data, and 70 were excluded due to the nonexistence of other data. After these exclusions, 5921 participants (2812 men and 3109 women) were eligible for the studies. Baseline assessments were obtained by face-to-face interviews or examinations. The questionnaires consist of demographic characteristics such as marital status and education years (low education means < 9 years, middle education means 9–12 years, high education means > 12 years) and physical activity (light < 3.0, intermediate 3.0–5.9, high ≥ 6.0) [21, 22], personal and familial medical history, and also smoking and the related data were collected by a standardized questionnaire [16, 22]. The Persian cohort study was approved by the Institutional Ethics Committee of Mashhad University of Medical Sciences (IR.MUMS.REC.1395.526; January 2017) and was conducted according to the guidelines of the Declaration of Helsinki. The informed consent was achieved from all participants before participation.

### 2.2. Clinical and Biochemical Analyses

At the baseline POCM examination, calibrated devices and standardized questionnaires were utilized by expert personnel (cardiologists, general practitioners, nurses, and dietitians) to collect all the data/information required by the study protocol [15, 20, 23, 24]. Weight (kilograms), waist and hip circumference (centimeter), height (meters), BMI (kg/m^2^), and anthropometric measurements were calculated using US National Institutes of Health protocols [25]. ABSI and BRI were calculated using the following formulas [5, 26]:

(1) $$\begin{array}{c} ABSI=\frac{WC}{\left(BMI2/3*height1/2\right)}\text{,}\\ BRI=364.2-365.5\times 1-\frac{WC}{2\pi 20.5}\text{.} \end{array}$$

The VAI was calculated based on the following sex-specific formulas [6]:

(2) $$\begin{array}{c} Males:VAI=\left(\frac{WC}{39.68+\left(1.88\times BMI\right)}\right)\times \left(\frac{TG}{1.03}\right)\times \left(\frac{1.31}{HDL}\right)\text{,}\\ Females:VAI=\left(\frac{WC}{39.58+\left(1.89\times BMI\right)}\right)\times \left(\frac{TG}{0.81}\right)\times \left(\frac{1.52}{HDL}\right)\text{.} \end{array}$$

Biochemical measurements were collected in a sitting position after 12 hours of fasting in the morning (BT1500 auto analyzer, Biotechnical Instruments, Rome, Italy). We used the standard methods of the World Health Organization (WHO) to measure fasting blood sugar (FBS), liver enzyme levels, serum lipid profiles, and other biochemical parameters. The Carotid-femoral pulse wave velocity (cf-PWV), used to evaluate arterial stiffness, was measured with the SphygmoCor XCEL System (AtCor Medical Incorporation) [27]. The test needs at least a 6 h fasting state and no caffeine, tobacco, and alcohol use for 12 hours prior to performing the test [28, 29]. The details of cf-PWV have been explained in previous studies [20, 27].

### 2.3. Statistical Analysis

Descriptive statistics of the study population were divided based on men and women and BMI (underweight <18.5 kg/m^2^, normal: 18.5–24.9 kg/m^2^, overweight: 25–29.9 kg/m^2^, and obese: 30 kg/m^2^). Continuous variables were expressed as mean ± standard deviation and discrete variables were expressed as a number and percentages. Differences among the groups were tested with a one-way analysis of variance (ANOVA) (continuous variables) or *χ*^2^ test (categorical variables). Multiple regression analysis was used to determine the association of anthropometrics variables on cf-PWV. The following factors were considered as independent variables: age, SBP, DBP, FBS, TG, and hypertension. All analyses were performed using SPSS software version 22, and *P* < 0.05 was considered statistically significant.

## 3. Results

A total of 5921 Persian adults (2812 men, 47.5%; 3109 women, 52.5%) were divided into four groups according to the BMI classification of the World Health Organization (WHO). The baseline characteristics such as demographic, anthropometric, arterial stiffness, laboratory findings, and clinical history are shown in Tables 1 and 2. Men had higher proportions of diabetes, age, cf-PWV, waist circumference, smoking, FBS, SBP, and DBP than women, but women had significantly higher VFA and hypertension than men. The mean VAI was 2.04 (underweight; *n* = 26), 3.38 (normal; *n* = 883), 4.51 (overweight; *n* = 1447), and 4.66 (obesity = 456) in men. The mean VFA were 48.9 in underweight, 98.5 in normal, 140.5 in overweight, and 187.3 in obese women.

Correlations between cf-PWV and the anthropometric measurements are shown in Table 3. In men with overweight, cf-PWV was significantly related to all indices: BMI (coefficient = 0.07, *P*=0.030), ABSI (coefficient = 0.14, *P* < 0.001), BRI (coefficient = 0.2, *P* < 0.001), WC (coefficient = 0.14, *P* < 0.001), VAI (coefficient = 0.08, *P* < 0.001), VFA (coefficient = 0.2, *P*=0.004), and WHR (coefficient = 0.15, *P* < 0.001). However, among men with obesity, cf-PWV was associated with BRI (coefficient = 012, *P*=0.009) VAI (coefficient = 1.01, *P*=0.009) and VFA (coefficient = 0.17, *P*=0.02). Among overweight women, cf-PWV was also related to all indices: BMI (coefficient = 0.14, *P* < 0.001), BRI (coefficient = 0.06 *P* < 0.001), WC (coefficient = 0.06, *P* < 0.001), VAI (coefficient = 0.16, *P* < 0.001), WHR (coefficient = 0.13, *P* < 0.001), and VFA (coefficient = 0.19, *P* < 0.001) except ABSI (coefficient = 0.003, *P*=0.91). Although, cf-PWV was only associated with VFA (coefficient = 0.186, *P* < 0.001), and WHR (coefficient = 0.17, *P* < 0.001) in women with obesity.

As shown in Table 4, multiple regression analysis was then performed to detect the variables that were strongly related to cf-PWV. The interaction between cf-PWV and anthropometric indices was influenced by age, SBP, DBP, FBS, TG, and hypertension in both men and women. Notably, the results were different between men with obesity and men with overweight. As shown in model III, cf-PWV was significantly associated with VAI (*β* = 0.54, *P* < 0.001), VFA (*β* = 0.07, *P* < 0.001), WC (*β* = 0.04, *P* < 0.001), and WHR (*β* = 0.08, *P* < 0.001) in men living with overweight. However, cf-PWV was only related to VAI (*β* = 0.65, *P* < 0.001) in men with obesity. In women living with overweight, cf-PWV was significantly associated with BMI (*β* = 0.06, *P* < 0.001), VFA (*β* = 0.10, *P* < 0.001), and WHR (*β* = 0.07, *P* < 0.001) in model III. Similarly, cf-PWV was significantly associated with VFA (*β* = 0.11, *P* < 0.001) and WHR (*β* = 0.05, *P* < 0.001) in women with obesity. Our results showed that VFA in women and VAI in men were the key factor that was strongly correlated with cf-PWV.

## 4. Discussion

In our cross-sectional study conducted in the population of 5921 Persian adults, after removing all confounding factors, we found that among the anthropometric indicators, VFA in women and VAI in men are related to arterial stiffness.

Arterial stiffness is known as a strong predictor of atherosclerosis, and as reported in various studies, atherosclerosis is strongly associated with cardiovascular events and mortality [30, 31]. Therefore, by evaluating the stiffness in the artery in patients and normal people, an estimate of cardiovascular events can be obtained.

In general, it has been seen that the use of anthropometric indices as new methods of evaluating central obesity and visceral obesity to determine the prognosis of arterial stiffness and subsequent cardiovascular events is reasonable [32].

In our study, in men with overweight, the arterial stiffness index was related to BMI-ABSI-BRI-WC-VAI-VFA-WHR and among men with obesity, a significant relationship was reported with BRI-VAI-VFA. Also, after modeling and removing confounding factors such as age, SBP, DBP TG, and hypertension, we came to the conclusion that arterial stiffness in overweight men is related to VAI-VFA-WC-WHR, and in men with obesity, it is only related to VAI. Also, in women with overweight, arterial stiffness index is related to BMI-BRI-WC-VAI-WHR-VFA criteria, and it is not related to ABSI. In women living with obesity, this index is related to VFA-WHR. But after modeling, it was found that in women with overweight, arterial stiffness is related to BMI-VFA-WHR, and in women with obesity, it is related to VFA-WHR.

BMI is one of the oldest measures to evaluate obesity in people; still, this measure does not differentiate between fat tissue and muscle mass [33, 34]. In addition, BMI does not distinguish the distribution of body fat in peripheral and abdominal areas [34, 35] although various studies have shown that fat deposits in the center and viscera are especially more harmful [36].

Several studies have reported that in Asian people, the distribution of fat, especially in the abdominal area, is more common than in European people. This factor is more common in Asian people than in European people with the same BMI and WC, despite the presence of metabolic syndrome and the risk of cardiovascular events [37, 38].

Also, in the study of Choi et al., it was reported that BMI is inversely related to arterial stiffness in men, and BMI, WC, and BRI in women are related to arterial stiffness, and this difference is due to the different distribution of fat in the body of women and men [39]. Because the majority of fat in men's bodies is visceral fat and subcutaneous fat in women's bodies. Considering the above, BMI is not a suitable factor for assessing the risk of cardiovascular events, especially in Asian people. In various studies, the role of BMI in relation to the evaluation of arterial stiffness has not been completely eliminated, but in various articles, new anthropometric factors have a greater and more important role in the evaluation of arterial stiffness in people with weight gain and obesity because in these new factors, the role of fat visceral is bolder and more important [16].

In the recent reports of the World Health Organization, it has been stated that WC is a substitute for BMI to determine the risk of diseases [40]. However, in Zhang et al.'s study, it was shown that WC has a weak relationship with arterial stiffness in women without the risk factors of diabetes and high blood pressure. It has an inverse relationship with arterial stiffness in men [32].

BMI and WC could not accurately represent abdominal visceral fat, so the newer ABSI and BRI measures were used, although these older measures of BMI, WC, and WHR were essential in previous studies to assess cardiovascular metabolic risk [40].

A valuable anthropometric index that was investigated in our study subjects was the ABSI index, which in our study was related to arterial stiffness with weight gain in men, but after removing the confounding factors, this factor was not significantly associated with arterial stiffness in any of the groups.

This index has been mentioned in various studies as an independent factor of weight height and BMI and it has been reported that it has a direct relationship with visceral fat and the risk of mortality and disease [1, 26]. In several studies, the relationship of this index with cancer mortality and cardiovascular disorders was compared with BMI, WC, and WHtR indices, and the relationship of this index was reported to be stronger and it was even accepted that this index is a marker of arterial stiffness in type 2 diabetes patients [1, 41] But later in several studies, it was seen that this index is a weaker index than BMI and WC for evaluating cardiovascular events and metabolic syndrome [42–44] Zhang et al. also concluded that in the population of Chinese people, this index is not a very good index for evaluating arterial stiffness [32].

In previous studies, it has also been reported that WHR, ABSI, and BRI have a strong relationship with arterial stiffness in both sexes, while BMI and WC have an inverse relationship with arterial stiffness. Also, two new anthropometric indices, ABSI and BRI, can predict Arterial stiffness indicators are valuable [44]. In this cross-sectional study, we found that new anthropometric indices had a significant relationship with PWV in both gender categories, while BMI and WC showed a negative relationship with PWV [32].

Another studied index is BRI, which has been shown in studies to be a better index than BMI and WC for evaluating body fat and visceral adipose tissue volume [5] But compared to other anthropometric indicators, not many studies have been conducted to assess the risk of metabolic syndrome and cardiovascular events. Only a few studies have described this index as valuable in evaluating cardiovascular events [44–46].

In our study, arterial stiffness in men with weight gain, men with obesity, and women with weight gain had a significant relationship with this index. In another published study, it was also reported that the BRI index with stiffness arterial correlates in the Chinese population [32].

One of the anthropometric indicators that had a significant relationship with arterial stiffness in both women and men in our study was the WHR factor, which measures the ratio of waist circumference to hip circumference. In the study of Zhou et al., it was reported that this factor has the best relationship with increased blood pressure in men [47].

WHR is one of the obesity parameters that are more related to abdominal obesity and has been shown in studies to be related to cardiovascular and cerebrovascular diseases [48].

In our study, this index was related to weight gain in men and in women with weight gain and in women with obesity and arterial stiffness and after removing the confounding factors of this WHR index with arterial stiffness in men with weight gain and women with weight gain and Obesity is related.

It was also seen in a systematic review that WHR is a suitable screening assessment for metabolic syndrome in adults [49], and this factor can determine the risk of cardiovascular events and metabolic syndrome in nonobese people [50]. This case was the same as the findings of the Zhang et al. study [32]. All these cases show that WHR is a suitable index for evaluating central obesity and can be a reliable measure in evaluating diseases related to central obesity, including metabolic syndrome and cardiovascular disorders.

Another anthropometric index is VAI, which was shown in our study to be very strongly related to arterial stiffness in men.

VAI is an index that shows the distribution of fat in the body based on BMI, WC, TG levels, and HDL levels. In the study of Yang et al., it was shown that this index could be a measure of obesity and assessment of atherosclerosis [51] And, in Cho et al. study and arterial stiffness in the Korean population, it was seen that VAI in men and women is related to arterial stiffness [39].

Another essential index in evaluations is VFA, which in our study was found to be strongly related to arterial stiffness in women. In the Li et al. study, it was also shown that this index has a direct relationship with arterial stiffness [16]. Also, in this study, it was shown that VFA in people with weight gain and obesity is related to arterial stiffness, and the relationship of this index with arterial stiffness is more potent than other indices [16]. Although in some previous studies, it was shown that the correlation of this index with arterial stiffness is weak [1].

In the Zajac et al. study, which investigated the relationship between VFA and metabolic syndrome in the female population, they concluded that metabolic syndrome in women is directly related to the VFA index [8]. In Zhang et al. study, they also concluded that the VFA index in women has a strong relationship with hypertension [7]. Several studies have reported that in Asians, the distribution of total body fat in the abdomen is higher and the incidence of CVD and metabolic risk factors are higher than in European people with the same BMI [37, 38] Therefore, BMI as a predictor of vascular disease or organic vascular dysfunction, it is not suitable especially for Asians [32].

Population-based cohort design, with a large number of participants, in Mashhad, the second largest city in Iran and, considering innovative Anthropometric indices in Persian adults are some of the strengths of the current study. The standard questionnaires consist of demographic characteristics, physical activity, personal and familial medical history, and smoking. Also, clinical and biochemical analyses may improve the judgment of the results. Using Pulse wave velocity (PWV), the safe, robust, reproducible, and noninvasive methods to evaluate arterial stiffness are strengths.

Although this study's findings may not be generalizable to other ethnicities, the results might still be applicable because of mostly consistent with previous studies around the world.

## 5. Conclusion

The result of the current study confirmed that the VFA index in women and the VAI index in men are strongly related to arterial stiffness. Therefore, the recommendation to improve these indices, such as lifestyle modifications and exercise habits developments, can improve arterial stiffness, which logically results in less cardiovascular disease.

## Figures and Tables

**Table 1:** Characteristics of participants according to BMI.

Variable	Men = 2812n						Women = 3109n					
	Under weight = 26	Normal = 883	Over weight = 1447	Obesity = 456	Total = 2812	*P*value	Under Weight = 21	Normal = 1057	Over weight = 1439	Obesity = 592	Total = 3109	*P*value
Age (years)	46.3 ± 13.6	46.9 ± 11.2	46.9 ± 10.5	47.6 ± 9.9	47.3 ± 10.67	<0.001	40.5 ± 9.6	41.1 ± 8.3	44.6 ± 8.9	46.7 ± 9.7	43.66 ± 9.14	<0.001
Education (years)												
Low (<9 y)	5 (0.2%)	72 (1.7%)	113 (4%)	35 (1.2%)	225 (8%)	0.14	1 (0.1%)	15 (0.5%)	40 (1.3%)	40 (1.3%)	96 (3.1%)	<0.001
Middle (9–12 y)	3 (0.1%)	233 (8.3%)	374 (13.3%)	120 (4.3%)	730 (26%)		2 (0.1%)	158 (5%)	317 (10.2%)	185 (6%)	662 (21.3%)	
High (>12)	18 (0.6%)	578 (20.5%)	960 (34.1%)	301 (10.7%)	1857 (66%)		18 (0.7%)	884 (28.1%)	1082 (34.9%)	367 (11.8%)	2351 (75.6%)	
Marital status n%												
Single	3 (0.1%)	34 (1.2%)	37 (1.3%)	8 (0.3%)	82 (2.9%)	0.06	8 (0.3%)	219 (7.0%)	147 (4.7%)	36 (1.2%)	410 (13.2)	<0.001
Married	22 (0.8%)	834 (29.7%)	1381 (49.1%)	443 (15.8%)	2680 (95.3%		10 (0.3%)	765 (24.6%)	1165 (37.5%)	485 (15.6%)	2425 (78%)	
Widowed	1 (0.01%)	8 (0.3%)	11 (0.4%)	1 (0.01%)	21 (0.7%)		0	16 (0.5%)	47 (1.5%)	28 (0.9%)	91 (2.9%)	
Divorced	0	7 (0.2%)	18 (0.6%)	4 (0.1%)	29 (1.1%)		3 (0.1%)	57 (1.8%)	80 (2.6%)	43 (1.4%)	183 (5.9%)	
Physical activity (mets n%)												
Low (<3.0 METs)	0	8 (0.3%)	13 (0.5%)	6 (0.3%)	27 (1%)	0.54	1 (0.01%)	8 (0.3%)	14 (0.5%)	4 (0.1%)	27 (0.9%)	0.27
Intermediate (3.0–5.9 METs)	23 (0.8%)	792 (28.5%)	1310 (47.1%)	423 (15.2%)	2548 (91.7%		18 (0.6%)	944 (30.9%)	1298 (42.4%)	519 (17%)	2779 (90.8%)	
High (≥6.0 METs)	2 (0.1%)	73 (2.6%)	106 (3.8%)	24 (0.9)	205 (7.4%)		2 (0.01%)	80 (2.6%)	112 (3.7%)	59 (1.9%)	253 (8.3%)	
Smoking (n, %)												
Never	24 (0.9%)	794 (28.5%)	1274 (48.7%)	401 (14.4%)	2493 (89.5%	0.87	21 (0.7%)	1040 (33.8%)	1417 (46%)	574 (18.6%)	3052 (99.1%)	0.87
Former	0	6 (0.2%)	7 (0.3%)	3 (0.1%)	16 (0.6%)		0	1 (0.1%)	0	0	1	
Current	2 (0.1%)	78 (2.8%)	150 (5.4%)	48 (1.7%)	278 (10.1%)		0	5 (0.2%)	11 (0.4%)	11 (0.4%)	27 (0.9)	
Hypertension (n, %)	0	62 (2.2%)	112 (4%)	42 (1.5%)	216 (7.7%)	0.001	0	40 (3.8%)	156 (10.9%)	127 (21.5%)	323	<0.001
Diabetes (n, %)	0	72 (2.6%)	207 (7.4%)	97 (3.5%)	376 (13.4%)	0.23	0	23 (0.7%)	64 (2.1%)	60 (1.9%)	147 (4.7%)	<0.001
SBP (mm Hg)	104.2 ± 12.2	108.1 ± 12.2	113.6 ± 14.5	118.8 ± 14.9	112 ± 14.6	<0.001	94.16 ± 12.1	97.7 ± 12.3	101.8 ± 13.6	107.45 ± 14	101 ± 13.8	<0.001
DBP (mm Hg)	64.5 ± 6.3	68.9 ± 8.3	72.7 ± 9.2	77.1 ± 9.60	72 ± 9.4	<0.001	62.4 ± 7.2	64.2 ± 7.5	66.7 ± 8.48	69.3 ± 8.8	66.2 ± 8.3	<0.001
MAP (mm Hg)	80.3 ± 6.9	86.32 ± 9.2	90.1 ± 10.1	94.7 ± 11.2	89.1 ± 11.6	<0.001	80.4 ± 7.1	83.5 ± 9.3	86.6 ± 9.9	91.1 ± 10.4	85.7 ± 10.1	<0.001
HR (bpm)	64.2 ± 11.8	65.2 ± 9.5	66.1 ± 9.3	68.4 ± 9.9	65.06 ± 9.3	<0.001	68.1 ± 5.9	68.6 ± 9.02	68.83 ± 9.08	69.8 ± 10.1	68.8 ± 9.2	0.24
Cf-PWV (cm/s)	6.05 ± 0.8	7.24 ± 1.47	7.70 ± 1.58	8.16 ± 1.72	7.5 ± 1.6	<0.001	5.93 ± 1.04	6.24 ± 1.22	6.78 ± 1.45	7.40 ± 1.73	6.6 ± 1.5	<0.001
TC (mg/dl)	161.8 ± 34.6	177 ± 35.7	181.4 ± 36.5	185.27 ± 39.6	180 ± 37.7	<0.001	171 ± 34.78	172.6 ± 36.1	181 ± 36.65	186.3 ± 37.15	178.2 ± 36.8	<0.001
TG (mg/dl)	80.8 ± 34.8	119.06 ± 70.8	152.4 ± 85.8	161.1 ± 83.4	142.5 ± 82.7	<0.001	74.8 ± 38.4	88.1 ± 48.05	108.55 ± 55.9	129.7 ± 66.27	104.6 ± 56.5	<0.001
LDL-C (mg/dl)	89.05 ± 25.1	99.1 ± 30.9	100.7 ± 32.7	102.42 ± 34.6	99.7 ± 31.2	<0.001	84.67 ± 29.7	92.5 ± 29.2	99.43 ± 30.45	102.6 ± 31.8	91.3 ± 30.4	<0.001
HDL-C (mg/dl)	56.8 ± 12.2	54.5 ± 12.16	50.6 ± 10.6	51.4 ± 10.8	52.2 ± 11.5	<0.001	71.94 ± 13.2	62.9 ± 13.7	60.24 ± 13.07	58.1 ± 12.4	60.4 ± 13.5	<0.001
ALT (U/L)	17.3 ± 6.4	25.6 ± 11.2	31.2 ± 13.7	35.2 ± 14.9	33.9 ± 8.6	<0.001	15.8 ± 4.3	18.4 ± 8.9	20.9 ± 9.9	23.8 ± 11.3	19.8 ± 7.1	<0.001
AST (U/L)	23.4 ± 13.7	22.1 ± 7.03	23.7 ± 7.8	25.68 ± 9.6	30.5 ± 14.2	<0.001	19.6 ± 4.3	18.9 ± 5.7	19.7 ± 7.2	21.4 ± 8.25	20.6 ± 10.1	<0.001
GGT (U/L)	22.7 ± 10.3	28.5 ± 20.5	32.5 ± 17.7	36.03 ± 15.1	31.2 ± 18.2	<0.001	18.17 ± 6.9	19.4 ± 13.6	22.7 ± 15.8	26.4 ± 21.07	22.2 ± 16.3	<0.001
BUN (mg/dl)	30.3 ± 4.7	32.6 ± 7.6	32.6 ± 7.6	32.4 ± 6.8	32.7 ± 7.6	0.563	28.8 ± 6.2	29.9 ± 6.6	27.6 ± 6.7	28.3 ± 6.9	27.7 ± 6.8	<0.001
Cr (mg/dl)	1.20 ± 0.18	1.22 ± 0.21	1.26 ± 0.23	1.2 ± 0.24	1.26 ± 0.22	<0.001	0.98 ± 0.15	0.96 ± 0.16	0.98 ± 0.1	1.01 ± 0.2	1 ± 0.18	<0.001
FBS (mg/dl)	91.4 ± 15.5	98.9 ± 26.4	102.5 ± 26.4	105.6 ± 27.1	101.4 ± 26.3	<0.001	88.8 ± 11.5	89.7 ± 17.9	93.9 ± 19.4	101.5 ± 27.8	91 ± 21.8	<0.001

SBP, systolic blood pressure; DBP, diastolic blood pressure; PWV, pulse wave velocity; MAP, mean arterial pressure; FBS, fasting blood sugar; TC, total cholesterol; TG, triglycerides. Data are presented as mean values and standard deviations or absolute and relative frequencies. *P* values derived from ANOVA for the normally distributed variables, and chi-square test for the categorical variables. The mean difference is significant at the 0.05 level.

**Table 2:** Anthropometric data of population by gender.

Variable	Men = 2812n					Women = 3109n				
	Under weight = 26	Normal = 883	Over weight = 1447	Obesity = 456	*P*value	Under Weight = 21	Normal = 1057	Over weight = 1439	Obesity = 592	*P*value
ABSI	0.86 ± 0.19	0.84 ± 0.11	0.82 ± 0.09	0.81 ± 0.09	0.009	0.87 ± 0.08	0.85 ± 0.1	0.84 ± 0.08	0.82 ± 0.09	0.006
BRI	2.55 ± 0.66	3.95 ± 0.75	4.92 ± 0.75	6.27 ± 1.21	<0.001	2.78 ± 0.7	4.35 ± 0.9	5.56 ± 1.11	7.32 ± 1.6	<0.001
VAI	2.04 ± 1.01	3.38 ± 2.82	4.51 ± 3.32	4.66 ± 3.04	<0.001	2.20 ± 1.3	3.1 ± 2.2	3.97 ± 2.5	4.83 ± 3.1	<0.001
BMI	17.16 ± 1.04	22.88 ± 1.56	27.25 ± 1.40	32.44 ± 2.42	0.006	17.7 ± 0.52	22.97 ± 1.45	27.19 ± 1.40	32.72 ± 2.77	0.007
VFA	29.17 ± 11.4	68.63 ± 18.91	104.38 ± 23.91	156.6 ± 32.6	<0.001	48.9 ± 8.26	98.5 ± 24.7	140.5 ± 24.6	187.3 ± 27.6	<0.001
WC	77.85 ± 8.26	91.36 ± 6.4	99.33 ± 5.64	109.3 ± 7.03	<0.001	75.9 ± 6.85	87.48 ± 7.64	95.61 ± 7.61	105.4 ± 9.47	<0.001
WHR	0.82 ± 0.02	0.90 ± 0.04	0.94 ± 0.05	1 ± 0.05	<0.001	0.83 ± 0.02	0.88 ± 0.04	0.92 ± 0.04	0.96 ± 0.05	<0.001

ABSI, a body shape index; BRI, body roundness index; VAI, visceral adipose index; BMI, body mass index; VFA, visceral fat area; WC, waist circumferences; WHR, waist-hip ratio.

**Table 3:** Correlations between PWV and anthropometric indices.

Variable	Men = 2812								Women = 3109							
	Under weight = 26		Normal = 883		Over weight = 1447		Obesity = 456		Under Weight = 21		Normal = 1057		Over weight = 1439		Obesity = 592	
	Coefficient	*P* value	Coefficient	*P* value	Coefficient	*P* value	Coefficient	*P* value	Coefficient	*P* value	Coefficient	*P* value	Coefficient	*P* value	Coefficient	*P* value
ABSI	−0.02	0.91	0.02	0.45	0.14	0.001	0.08	0.06	0.09	0.69	−0.04	0.11	0.003	0.91	−0.01	0.73
BRI	−0.1	0.63	0.15	0.001	0.20	0.001	0.12	0.001	0.06	0.76	0.03	0.3	0.06	0.001	0.05	0.20
VAI	−0.38	0.07	0.14	0.001	0.08	0.001	1.01	0.001	0.13	0.57	0.06	0.001	0.16	0.001	0.07	0.05
BMI	0.002	0.99	0.10	0.001	0.07	0.001	0.07	0.09	−0.05	0.81	0.12	0.001	0.14	0.001	0.07	0.08
VFA	0.023	0.91	0.17	0.001	0.2	0.001	0.17	0.001	−0.24	0.29	0.14	0.001	0.19	0.001	0.18	0.001
WC	−0.01	0.93	0.08	0.001	0.14	0.001	0.07	0.10	−0.005	0.9	0.01	0.64	0.06	0.001	0.04	0.24
WHR	0.17	0.39	0.11	0.001	0.15	0.001	0.059	0.20	0.08	0.73	0.11	0.001	0.13	0.001	0.17	0.001

**Table 4:** Comparison of the association of anthropometric measurements based on obesity status.

			Unadjusted			Model I			Model II				Model III			
			*β*	*R* ^2^	*P*value	*β*	*R* ^2^	*P*value	*β*	*R* ^2^	*P* value		*β*	*R* ^2^	*P* value	
Men	Underweight = 26	ABSI	0.07	−0.03	0.72	0.09	0.06	0.66	0.10	0.14		0.64	0.09	0.23		0.66
		BRI	−0.04	−0.04	0.88	−0.07	0.06	0.73	−0.05	0.15		0.81	−0.30	0.33		0.20
		VAI	−0.21	0.01	0.33	−0.26	0.15	0.19	−0.35	0.30		0.11	−0.38	0.28		0.45
		BMI	0.08	−0.03	0.68	0.05	0.05	0.77	0.18	0.17		0.36	0.13	0.24		0.50
		VFA	0.08	0.17	0.40	0.06	0.05	0.74	0.13	0.15		0.52	0.10	0.23		0.63
		WC	−0.04	0.03	0.87	0.08	0.06	0.67	0.10	0.15		0.62	−0.10	0.26		0.65
		WHR	0.21	0.06	0.30	0.28	0.13	0.15	0.32	0.24		0.12	0.21	0.27		0.31
	Normal = 883	ABSI	0.09	−0.01	0.80	−0.03	0.17	0.33	−0.02	0.21		0.38	−0.03	0.21		0.32
		BRI	0.18	0.03	<0.001	0.07	0.16	<0.001	0.06	0.19		<0.001	0.05	0.21		<0.001
		VAI	0.13	0.01	<0.001	0.12	0.18	<0.001	0.10	0.20		<0.001	0.03	0.21		0.71
		BMI	0.09	0.07	<0.001	0.11	0.18	<0.001	0.09	0.21		<0.001	0.07	0.22		<0.001
		VFA	0.20	0.04	<0.001	0.15	0.20	<0.001	0.13	0.21		<0.001	0.12	0.24		<0.001
		WC	0.13	0.01	<0.001	0.13	0.18	<0.001	0.11	0.20		<0.001	0.10	0.22		<0.001
		WHR	0.13	0.01	<0.001	0.16	0.20	<0.001	0.13	0.21		<0.001	0.11	0.23		<0.001
	Overweight = 1447	ABSI	0.07	0.05	<0.001	0.01	0.19	0.47	0.01	0.24		0.46	0.01	0.25		0.55
		BRI	0.17	0.03	<0.001	0.01	0.19	0.61	−0.02	0.24		0.93	−0.01	0.25		0.70
		VAI	0.04	0.02	<0.001	0.12	0.20	<0.001	0.11	0.24		<0.001	0.54	0.25		<0.001
		BMI	0.05	0.03	<0.001	0.03	0.19	0.11	0.01	0.23		0.50	0.01	0.25		0.52
		VFA	0.18	0.03	<0.001	0.09	0.20	<0.001	0.07	0.25		<0.001	0.07	0.26		<0.001
		WC	0.12	0.01	<0.001	0.06	0.19	<0.001	0.05	0.24		<0.001	0.04	0.25		<0.001
		WHR	0.13	0.01	<0.001	0.10	0.20	<0.001	0.09	0.24		<0.001	0.08	0.26		<0.001
	Obesity = 456	ABSI	0.08	0.05	0.07	0.05	0.10	0.24	0.05	0.14		0.21	0.05	0.14		0.22
		BRI	0.03	0.01	<0.001	0.06	0.11	0.15	0.07	0.14		0.12	0.07	0.15		0.10
		VAI	0.92	0.01	<0.001	0.04	0.11	<0.001	0.13	0.14		<0.001	0.65	0.14		<0.001
		BMI	0.01	0.02	0.70	0.01	0.10	0.18	−0.02	0.15		0.53	−0.03	0.14		0.51
		VFA	0.05	0.01	<0.001	0.02	0.10	<0.001	0.03	0.14		<0.001	0.04	0.14		0.45
		WC	0.01	−0.02	0.77	−0.06	0.10	0.95	−0.02	0.14		0.16	−0.03	0.14		0.52
		WHR	0.04	0.01	0.34	0.03	0.10	0.14	0.02	0.14		0.96	−0.08	0.13		0.85

Women	Underweight = 21	ABSI	0.11	−0.04	0.63	0.16	0.50	0.35	0.18	0.60		0.30	0.09	0.65		0.60
		BRI	0.06	−0.04	0.80	0.04	0.47	0.80	0.05	0.57		0.75	0.01	0.64		0.94
		VAI	0.38	0.10	0.86	0.17	0.50	0.33	0.24	0.63		0.14	−0.16	0.64		0.78
		BMI	−0.30	0.38	0.19	−0.37	0.62	<0.001	−0.37	0.74		<0.001	−0.30	0.72		0.09
		VFA	−0.35	0.08	0.11	−0.42	0.66	<0.001	−0.44	0.80		<0.001	−0.40	0.18		<0.001
		WC	0.03	−0.05	0.88	0.11	0.48	0.52	0.12	0.58		0.50	0.05	0.64		0.75
		WHR	0.10	−0.04	0.64	−0.24	0.52	0.19	−0.15	0.60		0.37	−0.18	0.68		0.26
	Normal = 1057	ABSI	−0.01	−0.01	0.96	−0.03	0.18	0.30	−0.06	0.12		0.83	−0.05	0.21		0.85
		BRI	0.07	0.04	<0.001	−0.01	0.18	0.54	0.01	0.21		0.57	0.01	0.22		0.70
		VAI	0.08	0.07	<0.001	0.02	0.18	0.45	−0.01	0.21		0.71	−0.16	0.22		<0.001
		BMI	0.11	0.01	<0.001	0.09	0.19	<0.001	0.08	0.22		<0.001	0.07	0.22		<0.001
		VFA	0.12	0.01	<0.001	0.08	0.18	<0.001	0.06	0.21		<0.001	0.05	0.22		<0.001
		WC	0.04	0.01	0.13	0.06	0.18	0.85	0.02	0.21		0.40	0.02	0.22		0.44
		WHR	0.08	0.07	<0.001	0.07	0.19	<0.001	0.04	0.21		0.11	0.04	0.22		0.11
	Overweight = 1439	ABSI	0.02	0.01	0.40	−0.02	0.15	0.38	0.01	0.18		0.99	−0.06	0.21		0.80
		BRI	0.1	0.09	<0.001	−0.09	0.15	0.73	−0.04	0.18		0.86	−0.02	0.21		0.40
		VAI	0.20	0.03	<0.001	0.12	0.16	<0.001	0.09	0.19		<0.001	−0.03	0.21		0.62
		BMI	0.13	0.16	<0.001	0.09	0.16	<0.001	0.07	0.19		<0.001	0.06	0.22		<0.001
		VFA	0.18	0.03	<0.001	0.11	0.16	<0.001	0.11	0.20		<0.001	0.10	0.22		<0.001
		WC	0.09	0.08	<0.001	0.03	0.15	0.18	0.04	0.18		0.09	0.02	0.21		0.33
		WHR	0.14	0.02	<0.001	0.10	0.16	<0.001	0.09	0.20		<0.001	0.07	0.22		<0.001
	Obesity = 592	ABSI	0.16	−0.01	0.70	−0.06	0.13	0.09	−0.07	0.15		0.07	−0.08	0.16		<0.001
		BRI	0.07	0.04	0.08	−0.03	0.12	0.45	−0.05	0.15		0.20	−0.07	0.16		0.06
		VAI	0.06	0.02	0.15	0.05	0.11	0.19	0.02	0.15		0.50	−0.05	0.16		0.53
		BMI	0.10	0.09	<0.001	0.08	0.13	<0.001	0.05	0.16		0.16	0.03	0.16		0.40
		VFA	0.16	0.02	<0.001	0.09	0.13	<0.001	0.06	0.15		0.10	0.11	0.16		<0.001
		WC	0.06	0.03	0.11	0.01	0.12	0.80	−0.04	0.15		0.92	−0.02	0.16		0.50
		WHR	0.13	0.01	<0.001	0.10	0.14	<0.001	0.06	0.15		0.11	0.05	0.16		<0.001

Model I: adjusted for age. Model II: model I + SB, DB. Model III: model II + FBS, TG, hypertension.

## Data Availability

The data used to support the study are included in the paper.
